# Validation of a small-size pooling approach targeting hospital surveillance of SARS-CoV-2 infection

**DOI:** 10.1017/ice.2020.380

**Published:** 2020-07-30

**Authors:** Andrea Petrucca, Marina Borro, Luana Lionetto, Giovanna Gentile, Antonella Alari, Maurizio Simmaco, Iolanda Santino

**Affiliations:** 1 Microbiology Unit, Sant’Andrea Hospital, Rome, Italy; 2 Department of Neurosciences, Mental Health and Sensory Organs, Sapienza University of Rome, Rome, Italy; 3 Sant’Andrea Hospital, Rome, Italy

*To the Editor*—The ongoing coronavirus disease 2019 (COVID-19) pandemic, caused by severe acute respiratory syndrome coronavirus 2 (SARS-CoV-2), started in December 2019 as a large viral pneumonia outbreak in the city of Wuhan, China.^1^ The disease spread from Wuhan to other countries, and the World Health Organization declared it a pandemic by March 11, 2020 (https://www.who.int/emergencies/diseases/novel-coronavirus-2019/events-as-they-happen). With vaccine development currently underway, the rapid identification of disease carriers and their close contacts represents the only effective measure to limit SARS-CoV-2 spreading.^2^

Hospitals are hotbeds for SARS-CoV-2 transmission; healthcare workers (HCWs) are at high risk of being infected and of further transmitting the virus to vulnerable patients.^3^ Thus, infection control strategies based on SARS-CoV-2 testing in HCWs and patients are necessary.^4^ Unfortunately, this type of disease surveillance is limited by the overwhelming demand for SARS-CoV-2 molecular diagnostic analyses.^3,5,6^

To increase COVID-19 testing capacity, procedures based on pooling of naso-oral pharyngeal (NOP) swab specimens have been recently proposed.^7–9^ However, the validation of the sample pooling approach is crucial to assess its diagnostic accuracy and to avoid false-negative results. Recent studies describing the detection of SARS-CoV-2 RNA in pools of 5 to 32 samples reported false-negative rates up to 10% for large groups, suggesting that smaller sample pools are a good compromise to increase sample processing capacity while maintaining test reliability.^6–9^ Since 5-sample pools were shown to efficiently detect SARS-CoV-2 RNA in RT-PCR assays,^7^ we chose to test and validate this approach using a high-throughput RNA extraction and amplification platform. The Sant’ Andrea Hospital of Rome (Italy) has put in place a SARS-CoV-2 surveillance program focused on the periodic screening of HCWs and preventive screening of patients (before hospitalization). In total, 2,035 people from the surveillance program (1,437 HCWs and 598 patients) were enrolled in this study. The molecular diagnostic workflow we used for SARS-CoV-2 detection included the following elements: (1) NOP swab sampling using the COPAN UTM-RM virus transport medium (Copan Diagnostics, Murrieta, CA); (2) automated specimen RNA extraction and amplification with the Versant kPCR molecular system (Siemens Healthineers AG, Erlangen, Germany). Viral nucleic acid detection was carried out using the detection kit for 2019 novel coronavirus (2019-nCoV) RNA (PCR-fluorescence probing; Daan Gene, Sun Yat University, Guangzhou, Guandong, China), an RT-PCR assay which simultaneously detects the viral nucleocapsid (N) and Orf1ab genes.

**Fig. 1.**
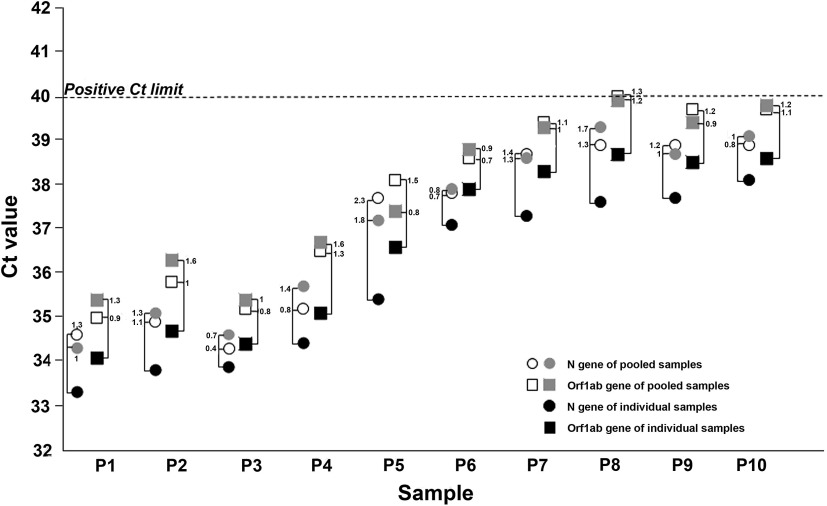
Influence of pooling samples strategy on the sensitivity of RT-PCR. Cycle threshold (Ct) values obtained from individual positive naso-oral pharyngeal swabs (NOP) samples (P1–P10; black symbols) and from their corresponding pooled samples, run in duplicate (open and grey symbols). Circles and squares indicate Ct values of the N and Orf1ab genes, respectively. Connecting brackets indicate the change in Ct (ΔCt) between individual NOP positive samples and their corresponding pools. The horizontal dotted line represents the Ct limit of our RT-PCR assay to assign a positive detection of SARS-Cov-2 RNA in NOP specimens.

We first tested a small set of NOP swab pools to assess the lower detection limit of the method, then we validated the method on a larger set of sample pools from Sant’ Andrea Hospital HCWs and patients. Each sample was analyzed both individually and as a part of a pool of 5 specimens (200 μL each). The small set consisted of 10 pools, each including 1 SARS-CoV-2–positive sample and 4 negative NOP samples. For each pool, 2 technical replicates were prepared and analyzed. The PCR cycle threshold (Ct) values of individually tested positive samples ranged from 33.3 to 38.1 for the N gene and from 34.1 to 38.7 for the Orf1ab gene, whereas Ct values obtained from their corresponding pools were between 34.3 and 38.9 for the N gene and between 35 and 40 for the Orf1ab gene (Fig. 1). The Ct value differences (ΔCt) between individual and pooled positive samples ranged from 0.3 to 2.3 for N and from 0.4 to 1.8 for Orf1ab (Fig. 1). No false-positive amplification signals were obtained using an analogous set of sample pools consisting of only SARS-CoV-2–negative NOP specimens.

We next performed a validation of the pooling strategy to assess the diagnostic performance and benefits of this approach. Daily during the first 3 weeks of April 2020, we analyzed an average of 96.9 individual NOP samples and their corresponding 19.38 pools collected from Sant’ Andrea Hospital HCWs and patients (2,035 individual samples and 407 pools). In total, 36 patients (1.7 %) were identified as SARS-CoV-2–positive through the analysis of individual samples as well as of their corresponding pools. Interestingly, all SARS-CoV-2–positive study participants belonged to the HCW group. In individually tested positive NOP specimens, the average Ct value for the N gene was 29.6 (±4.7) and the average Ct value for the Orf1ab gene was 31.1 (± 5.6). In pooled samples, the average Ct value for the N gene was 31.7 (±5.9) and the average Ct value for the Orf1ab gene was 33.8 (±6.1).

The diagnostic accuracy of the 5-sample pooling strategy was excellent, showing sensitivity, specificity, and positive and negative predictive values of 100%. The tests required to complete individual NOP sample and pool analysis were, respectively, 2,035 and 587 (407 pools plus 36×5 = 180 tests to confirm single samples included in positive pools). Summarizing, the small-pooling approach saved 1,448 tests, corresponding to 71.1% of the total cost of laboratory reagents required for individual sample analysis (ie, 15 RNA extraction and RT-PCR amplification kits). In our hands, it was possible to run at least 2 consecutive analytical sessions per day, allowing the reanalysis of individual samples from positive pools within 24 hours, which is a standard laboratory turnaround time for SARS-CoV-2 diagnostics in Italy.

When COVID-19 incidence is low, as in our study (below 2%), the small-pooling approach significantly reduces the use of laboratory resources and simultaneously increases the number of screened people. The number of positive pools to be reanalyzed increases in relation to SARS-CoV-2 incidence, consequently worsening TAT and cost–benefit ratio. In conclusion, the described approach represents an optimal strategy for surveillance programs in late pandemic phases when screening of a large population is needed.
